# Albumin Proteins as Delivery Vehicles for PFAS Contaminants into Respiratory Membranes

**DOI:** 10.1021/acsomega.3c06239

**Published:** 2023-11-09

**Authors:** Evan S. Pye, Shannon E. Wallace, D. Gerrard Marangoni, Alexander C. Y. Foo

**Affiliations:** Dept. of Chemistry, St. Francis Xavier University, 2321 Notre Dame Avenue, Antigonish B2G 2W5, Nova Scotia, Canada

## Abstract

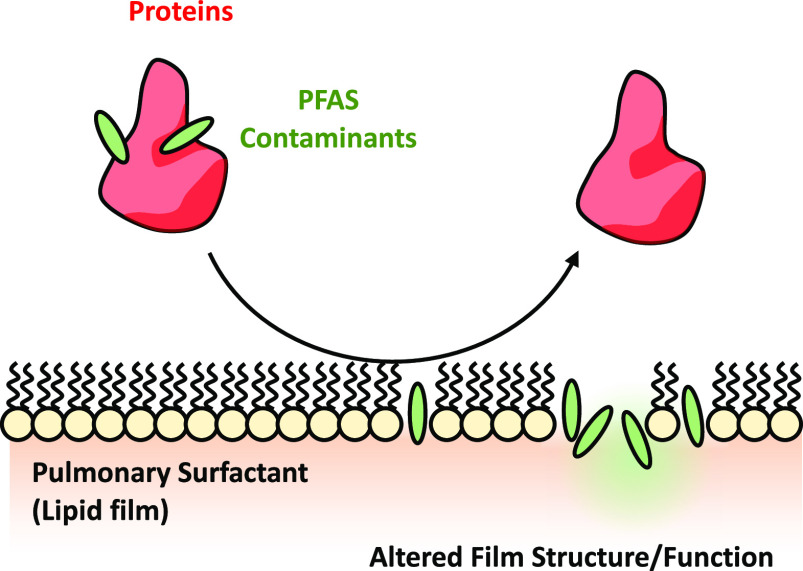

Poly- and perfluoroalkyl substances (PFAS) are a family of chemicals that have been used in a wide range of commercial products. While their use is declining, the prevalence of PFAS, combined with their chemical longevity, ensures that detectable levels will remain in the environment for years to come. As such, there is a pressing need to understand how PFAS contaminants interact with other elements of the human exposome and the consequences of these interactions for human health. Using serum albumin as a model system, we show that proteins can bind PFAS contaminants and facilitate their incorporation into model pulmonary surfactant systems and lipid bilayers. Protein-mediated PFAS delivery significantly altered the structure and function of both model membrane systems, potentially contributing to respiratory dysfunction and airway diseases in vivo. These results provide valuable insights into the synergistic interaction between PFAS contaminants and other elements of the human exposome and their potential consequences for human health.

## 1 Introduction

Poly- and perfluoroalkyl substances (PFAS) are highly fluorinated organic compounds that are used to produce a variety of industrial and consumer goods from building materials to furniture and cookware. The longevity of PFAS compounds, coupled with their widespread usage, has made them a ubiquitous environmental contaminant—with over 95% of adults from most first-world countries showing detectable levels of PFAS in their blood.^1,2^ Exposure to PFAS contaminants is skewed toward disadvantaged populations, with industrial pollution and contaminated buildings being major avenues of exposure.^2^ This exposure is correlated with an increased risk of respiratory allergies and other inflammatory disorders, contributing to health inequalities among effected populations.

The two PFAS most commonly found in the environment are perfluorooctanoic acid (PFOA) and perfluorooctanesulfonic acid (PFOS), both of which contain long (8-carbon) fluorinated acyl chains. Due to their long chains, PFOS/PFOA possess extremely high surface activities and readily adsorb onto an air–liquid interface. While this property contributes heavily to their industrial/commercial utility, it also enables them to absorb into and disrupt lipid membranes.^3^ The human respiratory system contains several lipid membranes. The outermost barrier is the pulmonary surfactant. Composed predominantly of dipalmitoylphosphatidylcholine (DPPC) lipids and supporting proteins, the pulmonary surfactant forms an ordered film (often termed a monolayer) at the air–liquid interface. Below the surfactant lie respiratory epithelial cells. The high surface activity and amphipathic nature of PFOA and PFOS could disrupt the structure and function of both of these lipid barriers, contributing to negative health outcomes.

PFOA and PFOS have low atmospheric volatility compared to short-chain PFAS. As such, dust particles represent both a major reservoir and an avenue of exposure for these compounds.^4,5^ Indeed, studies have reported levels over 1 μg/g of dust in some indoor environments,^5,6^ representing >60% of total airborne exposure.^4^ In addition to PFAS contaminants, these dust particles contain a variety of components including proteins—many of which tend to be allergens from common indoor sources such as dust mites or pet dander. These proteins are present on the μg/g scale^7^ and can potentially act as carriers for hydrophobic molecules such as PFOA/PFOS, facilitating their delivery and incorporation into respiratory membranes.

Albumins are abundant globular proteins which are able to bind a variety of compounds in a nonspecific manner, making them an ideal model system to study PFAS–protein interactions. In this work, we show that bovine serum albumin (BSA) can bind both PFOS and PFOA and deliver them into model lipid films at the air–liquid interface. Protein-mediated PFAS delivery significantly altered the surface activity of these lipid systems, destabilized their physical structure, and enhanced their susceptibility to hydrolysis by phospholipase A_2_ (PLA_2_) enzymes. BSA–PFAS complexes also showed activity against phospholipid bilayers, being able to permeabilize and lyse lipid vesicles. These studies suggest that environmental proteins could act as carriers to facilitate the delivery of long-chain PFAS into the respiratory environment and enhance their ability to destabilize both the pulmonary surfactant and the underlying epithelial cell membranes.

## 2 Materials and Methods

### 2.1 Preparation and Characterization of BSA–PFAS Complexes

BSA (Sigma, lyophilized powder ≥96%) was dissolved in phosphate-buffered saline (PBS, pH = 7.4) to a final concentration of 1.5–2.5 mM. PFOA or PFOA (Sigma, 95 and 98%, respectively) was added to achieve a final mole-ratio of at least 20:1 PFAS per BSA molecule. This significantly exceeds the binding stoichiometries observed for fatty acids and other structurally similar ligands and is intended to maximize binding.^8−10^ The BSA–PFOA/PFOS mixture was incubated at 37 °C overnight. The resulting protein–PFOA/PFOS complexes were then exchanged into PBS using a centrifugal filter unit (Amicon), with three complete rounds of buffer exchange to ensure the removal of excess (unbound) PFAS. The resulting protein was quantified using a BSA assay (Pierce).

To assess PFOA/PFOS binding stoichiometries, samples were diluted 1:1 with SDS buffer (PBS, 250 mM SDS) to completely dissolve the BSA–PFOA/BSA–PFOS complexes. PFOA/PFOS levels in the samples were assessed by using a Bruker AVANCE II 400 MHz NMR spectrometer. ^1^H-coupled ^19^F NMR spectra (Bruker pulse zgflqn) were obtained at 25 °C. The resulting spectra were similar to those reported in previous studies (S1).^11^ In both spectra, the peaks corresponding to the terminal CF_3_ fluorine nuclei were markedly separated from the CF_2_ peaks, making them ideal for quantitation. The intensities of these peaks were compared to those obtained from a standard curve (S1) generated using known concentrations of PFOS or PFOA diluted 1:1 in a PBS/SDS buffer.

### 2.2 Surface Tension Measurements

Surface tension measurements were obtained by using a LAUDA TD1C surface tensiometer equipped with a platinum-iridium Du Noüy Ring (Kruss Scientific) at room temperature. To prepare the aqueous subphase, 15 mL of PBS was added to a 50 mm dish. DPPC lipids (Avanti) were dissolved in methanol to a final concentration of 500 μg/mL. 75 μL of the DPPC-methanol stock was deposited on top of the aqueous subphase for a final loading of 2.5 μg/mL. This was sufficient to completely coat the surface of the air–liquid interface (S2). The lipids were incubated at room temperature for 10 min to allow complete evaporation of methanol and deposition of the DPPC film prior to surface tension measurements. Analyte (BSA, BSA–PFOA, BSA–PFOS, free PFOS, or free PFOS) was injected into the aqueous subphase and allowed to equilibrate for a further 10 min prior to surface tension measurement.

### 2.3 Lipid Film Destabilization

A DPPC-LAURDAN stock containing 50 μg/mL DPPC and 5.5 μg/mL LAURDAN was prepared in methanol. 150 μL of PBS containing the desired analyte was loaded onto a 96-well plate to generate the aqueous subphase. 7.5 μL of DPPC-LAURDAN was deposited on top of the aqueous phase and incubated for 10 min at 37 °C to allow for complete evaporation of the solvent and deposition of our lipid onto the air–liquid interface. LAURDAN fluorescence spectra were measured using a Spectramax M3 (Molecular Devices) 96-well plate reader at 37 °C with an excitation wavelength of 385 nm and an emission wavelength window of 400–550 nm. Generalized polarization (GP) was calculated by comparing the fluorescence intensity of the solid- and liquid-phase emission peaks as described previously.^12^ Spectra obtained using LAURDAN-DPPC in the absence of an analyte at 55 °C were used as a positive control to represent a DPPC monolayer that has completely transitioned to the liquid phase.

### 2.4 Phospholipase Activity

Phospholipase activity was assessed using the EnzChek PLA_2_ assay kit (Invitrogen) with some modifications to accommodate the monolayer environment. Substrate solution containing 10 μg/mL of the kit-provided EnzChek PLA_2_ substrate and 50 μg/mL of DPPC lipids was prepared in methanol. To generate the aqueous subphase, 150 μL of the kit provided 1× EnzChek reaction buffer containing the desired analytes along with 1.56, 3.12, or 4.68 U/mL porcine PLA_2_ (Sigma) was added to a 96-well plate. The reaction was initiated by depositing 7.5 μL of the substrate solution on top of the subphase. Substrate cleavage was monitored by observing the fluorescence intensity at 515 nm (excitation 460 nm) using a Spectramax M3 multimode plate reader (Molecular Devices) and plotted over time to yield a rate of reaction.

### 2.5 Vesicle Leakage

Calcein-loaded DPPC vesicles were generated as described previously.^13^ In brief, DPPC lipids were deposited onto a glass test tube. The lipids were heated to above the DPPC phase-transition temperature and resuspended in 0.27 mM NaOH solution containing 70 mg/mL calcein to generate large multilamellar vesicles. These vesicles were then extruded through a 1.0 μm membrane using a lipid extruder (Avanti) to generate uniformly sized small unilamellar vesicles (SUVs). SUVs were then cooled to below the DPPC phase-transition temperature. Calcein-loaded SUVs were recovered using centrifugation (15,000*g* for 3 min). The supernatant containing excess calcein was removed and replaced with fresh PBS. This buffer exchange was repeated 3–5 times to ensure complete removal of excess calcein.

Vesicle leakage was assessed using established protocols.^13^ Here, calcein-loaded DPPC vesicles (0.25 mg/mL) were incubated with the desired analyte for 30 min. Fluorescence intensity was measured at 530 nm with an excitation at 485 nm using a Spectramax M3 multimode plate reader (Molecular Devices). Vesicles exposed to PBS and 5% SDS were used as positive and negative controls, representing 0 and 100% vesicle lysis, respectively. All experimental values were normalized against these controls to obtain *a* % lysis value.

## 3 Results and Discussion

### 3.1 BSA Forms Stable Complexes with PFOA and PFOS

In this study, the PFOA/PFOS–BSA complex was prepared under aqueous conditions. The resulting PFOA/PFOS–BSA complexes displayed a binding stoichiometry of 4.5 ± 0.5 ligands per BSA molecule following multiple rounds of buffer exchange to remove unbound ligands (see Materials and Methods—Section 2.1). These findings are consistent with previous results showing that BSA and other serum albumins bind long-chain PFAS with stoichiometries of ∼1–4 and *K*_d_ values in the low micromolar range.^8−10^ Taken together, these results suggest that BSA forms extremely stable complexes with both PFOA and PFOS that are capable of protecting its bound ligands from the external environment.

### 3.2 BSA Mediates Delivery of PFOA/PFOS into Surface-Deposited Lipid Films

**Figure 1:**
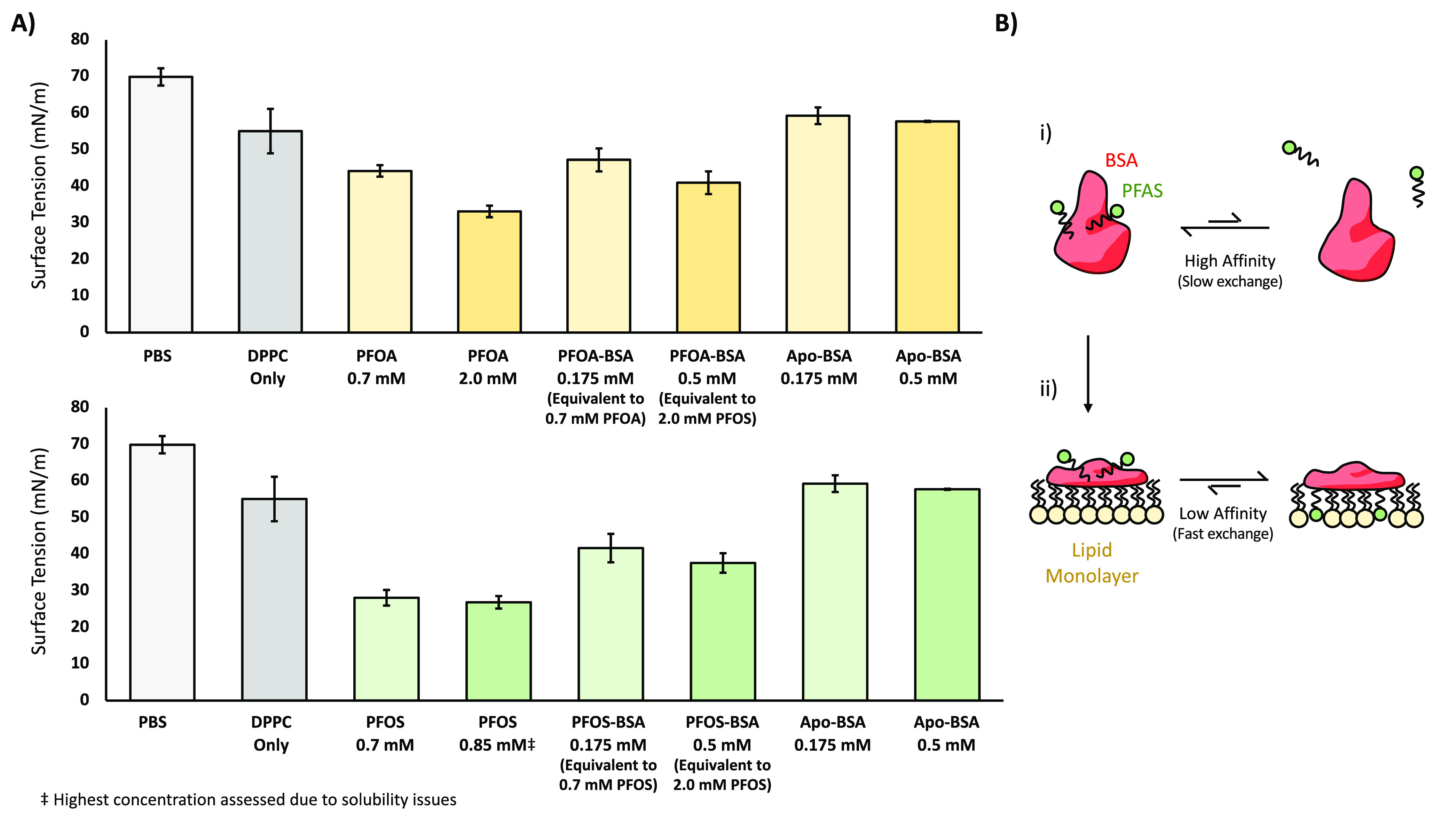
BSA-mediated PFAS delivery into DPPC monolayers: (a) impact of free PFOA/PFOS and BSA–PFOA/PFOS on the surface tension of a DPPC phospholipid film deposited onto an air–liquid interface of PBS. Analyte was injected into the aqueous subphase, and surface tension measured after a 10 min wait period. The surface tension of PBS with no lipids is shown for reference. The concentration of BSA used was adjusted to provide an equivalent concentration of PFOA/PFOS as the free PFOA/PFOS samples when possible, assuming a 4:1 binding stoichiometry. Reported values and error bars represent the mean and standard deviation taken from at least three replicates. (b) Model of BSA-mediated PFAS delivery. (i) BSA–PFAS complex in an aqueous environment binds PFAS with high affinity. This produces a stable, long-lived complex which experiences minimal ligand dissociation. (ii) BSA–PFAS complex undergoes a conformation change upon adsorption to a surfactant surface. This reduces its affinity for PFAS, facilitating rapid dissociation and delivery on the minute timescale.

DPPC is the predominant lipid component of the pulmonary surfactant. DPPC monolayers/films deposited onto an air–liquid interface thus represent a reproducible, well-defined model system to study the pulmonary surfactant. Previous studies showed that the addition of free PFOS and PFOA to the aqueous subphase of both whole pulmonary surfactant and synthetic phospholipid monolayers significantly reduced surface tension, with a lowest effective concentration of 0.5 mM.^14^ We were able to replicate these results under our experimental conditions with a significant reduction in surface tension being observed upon exposure of our DPPC monolayers to both PFAS at similar concentrations (Figure 1). To assess the impact of our BSA complexes, 0.175 mM of BSA–PFOA or BSA–PFOS was added to the aqueous subphase. For the sake of simplicity, a binding stoichiometry of ∼4:1 PFOA or PFOS per BSA molecule was assumed based on the results from the previous section. Thus, the concentration of BSA employed (0.175 mM) yields an equivalent of 0.7 mM PFAS. A similar reduction in surface tension was observed under these conditions. This perturbation was dependent on the presence of the PFOA/PFOS ligand as an equivalent concentration of Apo (empty) BSA actually yielded a change in the opposite direction. Increasing the concentration of analyte to 2 mM PFOA/PFOS (or equivalent) enhanced the magnitude of the effect, indicating that the observed phenomenon was dosage dependent. Taken together, these results suggest that BSA effectively transfers long-chain PFAS ligands into DPPC monolayers, altering their surface activity in a manner akin to their free PFOA/PFOS counterparts. It should be noted that this exchange occurs on the minute timescale (see Materials and Methods Section). This stands in marked contrast to the low *K*_d_ and high stability of the BSA–PFAS complexes under aqueous conditions—being able to withstand multiple rounds of buffer exchange as noted in Section 3.1. Previous studies show that serum albumins such as BSA readily absorb onto surfactant monolayers.^15,16^ This is accompanied by a conformation change, which could facilitate incorporation of any bound ligands directly into the lipid envelope of its target monolayer without dissociation of free PFAS into the aqueous phase (Figure 1).^17^ Such a model would reconcile the high affinity and stability of the aqueous BSA–PFAS complex with the fast PFAS delivery observed in the presence of a model lipid membrane. This phenomenon is not restricted to serum albumins as many common environmental proteins such as caseins, globulins, seed-storage proteins, and nonspecific lipid-transfer proteins (nsLTP) will also undergo conformation changes upon adsorption onto surfactant monolayers.^18−20^ These proteins are commonly found in house dust alongside PFAS contaminants. Additionally, many of these proteins are known to bind lipid ligands, suggesting that protein-mediated PFAS binding and delivery could be a widespread phenomenon.

### 3.3 BSA-Mediated PFAS Delivery Destabilizes Lipid Monolayers

**Figure 2:**
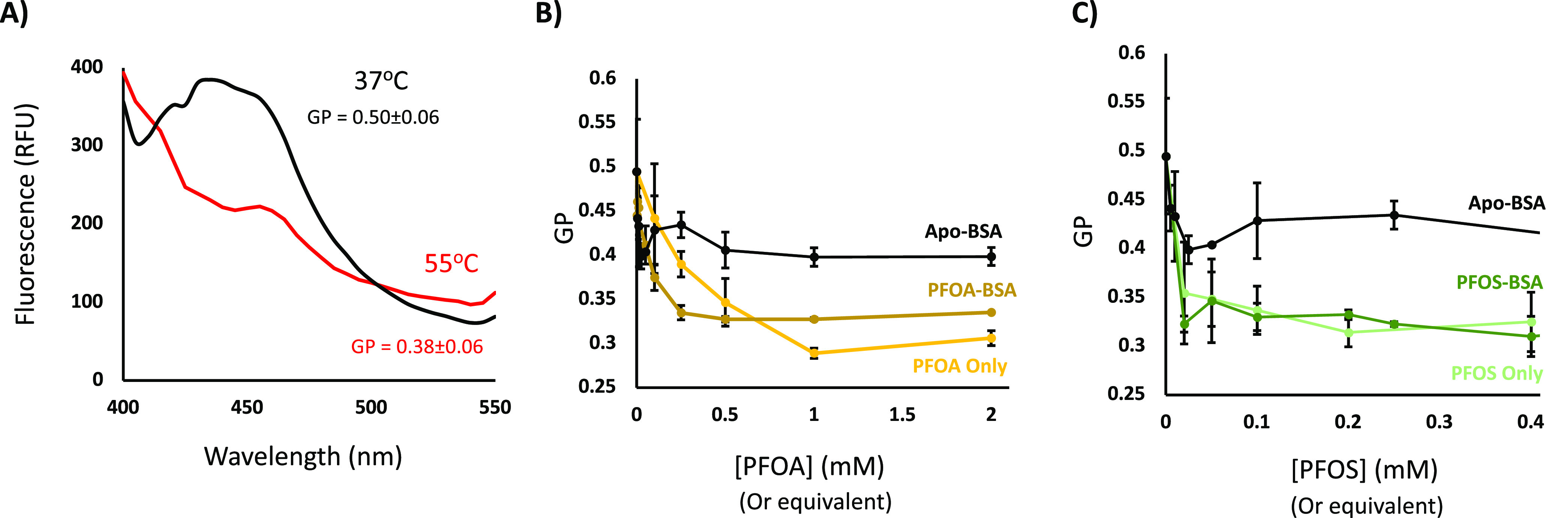
BSA-mediated PFAS delivery increases the fluidity of lipid monolayers. (a) Representative LAURDAN fluorescence spectra (Ex: 385 nm) taken for a DPPC lipid film at 37 and 55 °C. Given that the phase-transition temperature of DPPC is 42 °C, these spectra are taken to represent DPPC in the solid phase and liquid phase, respectively. The calculated GP values are shown. (b) GP values of DPPC films at 37 °C in the presence of increasing concentrations of PFOA, PFOA–BSA, or Apo-BSA. Concentrations of PFOA–BSA and BSA have been corrected assuming a binding stoichiometry of 4 PFOA/BSA (c) GP values of DPPC films at 37 °C in the presence of increasing concentrations of PFOS, PFOS-BSA, or Apo-BSA. Concentrations of PFOA–BSA and BSA have been corrected assuming a binding stoichiometry of 4 PFOS/BSA. Reported GP values and error bars represent the mean and standard deviation taken from at least three replicates.

To further assess the impact of BSA-mediated PFAS delivery, we examined the ability of our BSA–PFOA/PFOS complexes to disrupt the physical structure of lipid films. Here, we incorporated LAURDAN into the DPPC model system. Heating the lipid films above their phase-transition temperature induced a transition from the solid to the liquid phase. This is accompanied by a reduction in the intensity of the main LAURAN fluorescence peak at ∼450 nm (Figure 2), which can be expressed in terms of GP. Addition of free PFOA or PFOS induced a similar reduction in GP values (Figure 2) with lowest effective concentration values <0.5 mM—broadly matching those reported in the literature.^3,14^ Previous studies show that serum albumins are capable of disrupting surfactant monolayers.^15,16^ However, this effect is magnified in the presence of PFOA/PFOS ligands, suggesting that BSA-mediated PFAS delivery contributes to monolayer destabilization.

The incorporation of exogenous lipids can disrupt surfactant films and has been correlated with respiratory distress and lung disease.^21,22^ Delivery of long-chain PFAS into surfactant monolayers via BSA and other protein scaffolds could induce a similar effect. While the direct adsorption of serum albumins and other proteins, including environmental allergen such as Ole e 1 and Ole e 7 (olive pollen) can disrupt the structure and function of surfactant monolayers,^15,16,19,20^ the potential for these proteins to act as delivery vehicles for PFAS and other harmful environmental contaminants represents a novel approach and could potentially contribute to the negative health outcomes associated with exposure to both types of environmental contaminants.

### 3.4 BSA-Mediated PFAS Delivery Facilitates Digestion of Surfactant Lipids by Endogenous Phospholipase Enzymes

**Figure 3:**
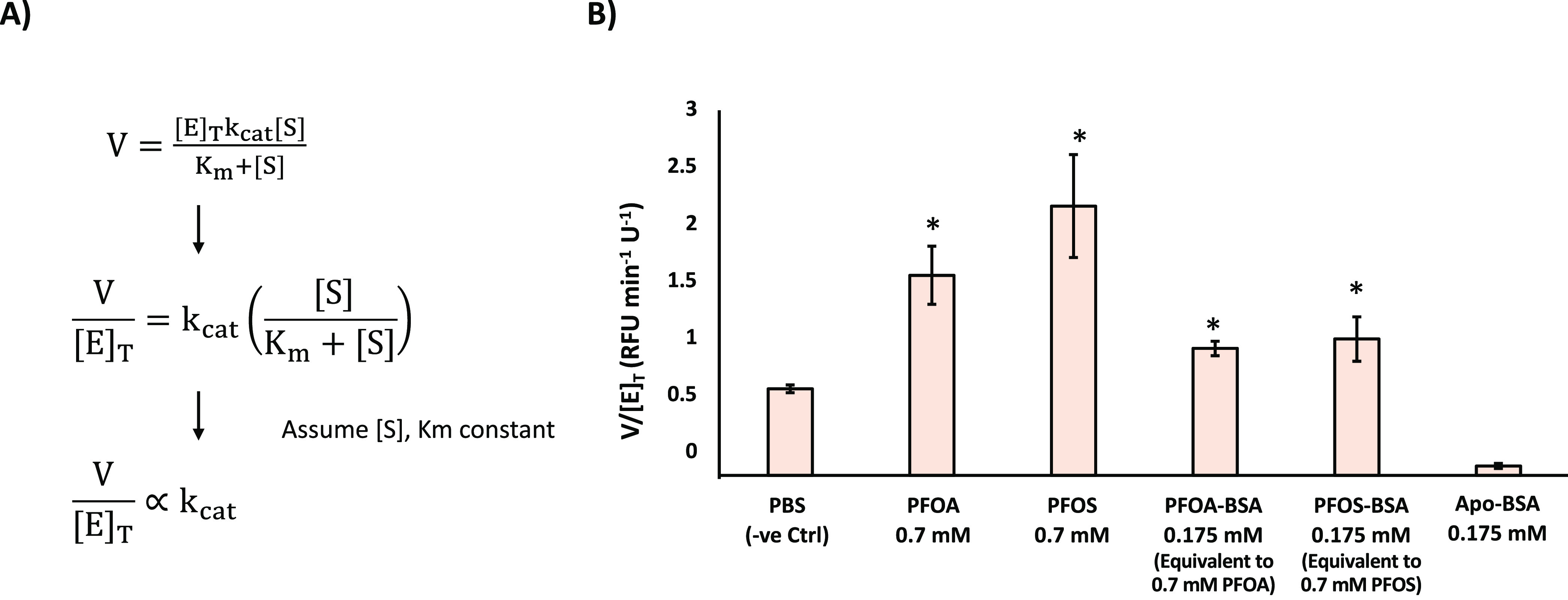
BSA-mediated PFAS delivery enhances susceptibility of surfactant lipids to degradation by PLA_2_ enzymes. (a) Rearrangement of the traditional Michaelis–Menten equation to show the relationship between *V*/[*E*]_*T*_ and *k*_cat_ (assuming constant [S] and *K*_m_). (b) V/[E]_T_ values for PLA_2_ against surface-deposited DPPC lipids in the presence of free- and BSA-bound PFAS. Reported values and error bars represent the mean and standard deviation taken from at least three replicates.* indicates values which differ statistically from the negative control (PBS) with a *p*-value < 0.05 as assessed via Student’s *t*-test.

Phospholipase A_2_ (PLA_2_) enzymes catalyze the breakdown of phospholipids, including those which make up the pulmonary surfactant. Normally, this process is tightly regulated, though increased PLA_2_ activity has been correlated with a variety of respiratory ailments.^23−25^ The rate-limiting step for most mammalian phospholipases is extraction of their phospholipid substrates into their active site.^26^ The surfactant-destabilizing properties of our BSA–PFAS complexes could facilitate this process, contributing to pulmonary surfactant degradation and its associated health consequences. To assess this hypothesis, we generated DPPC films incorporating a fluorogenic PLA_2_ substrate. Addition of mammalian PLA_2_ to the aqueous subphase resulted in the cleavage of the substrate, which could be quantified over time to generate a rate of reaction (*V*). Rates were measured using a final enzyme concentration ([*E*]_T_) of 2.5, 5.0, and 7.5 μg/mL, corresponding to 1.56, 3.12, and 4.68 U/mL of PLA_2_ activity, respectively. The slope of the resulting graph (*V*/[*E*]_t_) provides an indirect measurement of *k*_cat_ assuming substrate concentrations ([*S*]) and the Michaelis constant (*K*_m_) remain constant (Figure 3). Addition of 0.175 mM BSA–PFAS to the aqueous subphase yielded an almost 2-fold enhancement of *V*/[*E*]_*t*_ (and thus *k*_cat_). A similar enhancement was observed for the equivalent concentrations of free PFOA and PFOS (0.7 mM). Curiously, the Apo form of BSA inhibited PLA_2_ activity, suggesting the enhancements observed for the BSA–PFAS complexes are a product of BSA-mediated PFAS delivery, rather than any innate property of the BSA protein scaffold itself.

The human respiratory system includes several secreted PLA_2_ enzymes that are active within the lungs. Bronchoalveolar lavage fluid (BALF) taken from patients with inflammatory lung disorders shows an increase in the expression and activity levels of these secretory PLA_2_ enzymes. This is accompanied by a decrease in the levels of pulmonary surfactant lipids and a corresponding increase in their PLA_2_ hydrolysis products, giving rise to a model in which PLA_2_-mediated surfactant degradation contributes to respiratory illness and inflammation.^23,24^ In addition to directly degrading the pulmonary surfactant, increased PLA_2_ activity can facilitate the generation of pro-inflammatory lipid mediators such as prostaglandins and leukotrienes, further contributing to the development of inflammatory airway disease.^27,28^ The increase in PLA_2_ activity observed in our studies could induce a similar effect in vivo, further contributing to the negative health outcomes associated with exposure to both PFAS contaminants and environmental proteins.

### 3.5 BSA-Mediated PFAS Delivery Permeabilizes Lipid Bilayers

**Figure 4:**
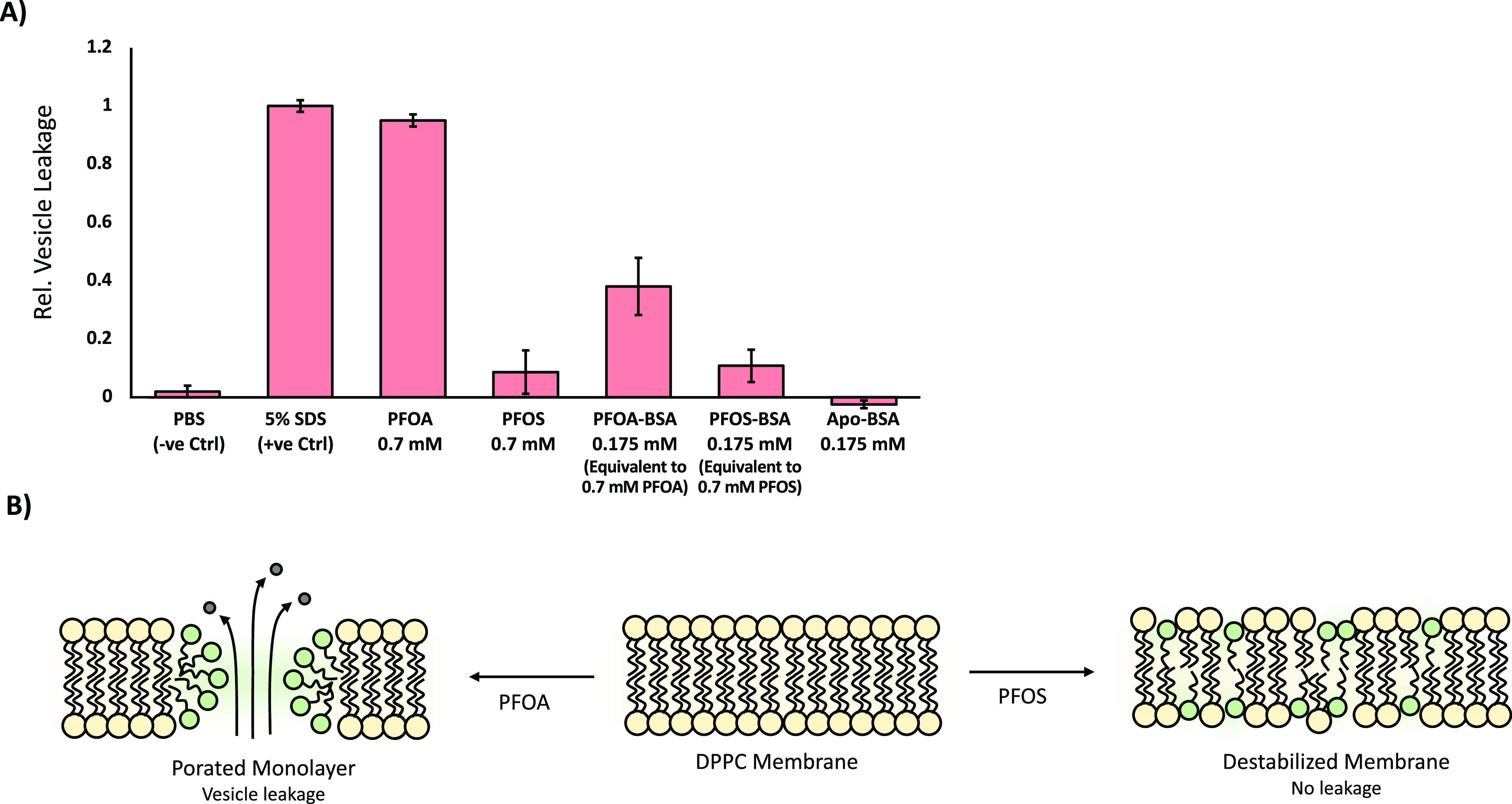
BSA-mediated PFAS delivery permeabilizes phospholipid bilayers. (a) Impact of free and BSA-bound PFAS on vesicle leakage as assessed using calcein fluorescence. Values normalized against negative (PBS) and positive (5% SDS) controls, representing 0 and 100% vesicle lysis, respectively. Reported values and error bars represent the mean and standard deviation taken from at least three replicates. (b) Potential model accounting for the reduced lytic activity of PFOS relative to PFOA. Here, the negative charge on the PFOS sulfate headgroup prevents aggregation within the membrane, resulting in the general dispersion of PFOS into the bulk membrane rather than the formation of concentrated pores.

The same properties which enable long-chain PFAS such as PFOA and PFOS to destabilize surfactant systems also allows them to lyse or permeabilize lipid bilayers, such as those found in the plasma membranes of respiratory cells.^29^ Using calcein-loaded lipid vesicles, we showed that PFOA is extremely effective at inducing vesicle leakage (Figure 4). This activity was retained when loaded onto its BSA scaffold, further highlighting the ability of protein scaffolds such as BSA to facilitate PFOA delivery. Curiously, PFOS—both in its free form and bound to BSA—displayed markedly reduced lytic activity. This contrasts with previous studies which show similar membrane-partitioning and destabilizing effects for both PFOA and PFOS in their free form. It is possible that the anionic nature of the PFOS headgroup prevents it from aggregating within the DPPC membrane due to charge repulsion, though further studies would be needed to confirm this conjecture (Figure 4). As with the surfactant experiments above, this transfer occurs on the minute timescale probably mediated by a lipid-bound BSA intermediate. While serum albumins readily partition to air–liquid interfaces and disrupt surfactant films,^17^ the same cannot be said for lipid bilayers. The lack of such an interaction could inhibit the formation of the lipid-bound BSA intermediate, potentially explaining the reduced potency of PFOA relative to the free PFAS. Nonetheless, BSA–PFOA retains significant membrane lytic activity relative to the negative control. When directed against the plasma membrane of epithelial cells, this lytic activity could activate damage-associated molecular patterns (DAMPs). These are pro-inflammatory compounds such as interleukin 1α (IL-1α), heat-shock protein 70 (HSP70), and tumor necrosis factor alpha (TNFα) which are released in response to cellular damage or stress.^33−35^ Damage to epithelial cells through the introduction of membrane-destabilizing agents can induce the upregulation of DAMPs,^36,37^ which can be correlated with asthma and other inflammatory disorders.^33−35^ The ability of BSA–PFOA complexes to permeabilize lipid bilayers could induce DAMP release in a similar manner, providing an additional avenue through which exposure to dust particles containing both PFAS and protein contaminants contributes to negative health outcomes among effected populations.^30−32^

In this work, we show that BSA can bind and deliver PFOA and PFOS into both model surfactant films and lipid bilayers disrupting their structure and function. It should be noted that PFAS contaminants are capable of inducing similar disruptions in the absence of BSA. However, the use of a protein scaffold has the potential to greatly enhance their solubility and bioavailability. For instance, in this work, we were able to achieve concentrations of BSA–PFAS in the 3 mM range: equivalent to over 12 mM of free-PFAS when taking into account the observed stoichiometric binding ratios. This exceeds the solubilities of both PFOS (∼2 mM) and PFOA (∼8 mM) under our experimental conditions. The use of a protein scaffold could also target PFAS to lipid membranes. As noted in Section 3.2, the stability and longevity of the PFAS–BSA complex under aqueous conditions contrasts with its rapid delivery into lipid membranes, suggesting the presence of a partially unfolded intermediate species (Figure 1). In the context of the human exposome, this model would ensure that PFAS remain sequestered within its protein scaffold and thus insulated from loss or degradation by the outside environment until it is exposed to a pulmonary surfactant or epithelial cell membrane, further increasing the efficacy of BSA and other lipid-binding proteins as a delivery vehicle for PFAS contaminants. Previous studies have attempted to leverage these same properties to design novel drug-delivery systems based on serum albumins and other lipid-binding protein scaffolds.^38−40^ Likewise, the use of albumins as a bioremediation tool to bind and sequester PFAS from the environment has also been explored.^41^ However, the ability of proteins to facilitate the delivery of PFAS into lipid membranes represents a novel pathway through which these compounds can interact with other elements of the human exposome to negatively impact human health.

The respiratory exposome contains numerous lipid-binding proteins such as 2S albumins and nsLTP’s from plant pollens, lipocalins from pet dander (Fel d 1, Can f 7), and other miscellaneous lipid carriers from cockroaches and dust mites (Bla g 1, Der p 5). These proteins (including BSA and other serum albumins under the correct circumstances) are important environmental allergens, exposure to which is correlated with both allergic airway disease and other inflammatory disorders.^42−44^ Given the prevalence of both lipid-binding allergens and PFAS contaminants in house dust and other airborne particulate matter,^45^ it is worth considering whether the model of protein-mediated PFAS binding and respiratory membrane disruption described in this work could contribute to the elevated risk of asthma and other inflammatory airway disease associated with exposure to both compounds.

## Supporting Information Available

NMR spectra of PFOS and PFOA, standard curves used to determine the binding stoichiometry of our BSA-bound complexes, and surface tension vs DPPC phospholipid graph confirming that our model DPPC films provide complete coverage of the air–liquid interface under our experimental conditions (PDF)

## References

[ref1] Lewis R.; Johns L.; Meeker J. Serum Biomarkers of Exposure to Perfluoroalkyl Substances in Relation to Serum Testosterone and Measures of Thyroid Function among Adults and Adolescents from NHANES 2011–2012. Int. J. Environ. Res. Public Health 2015, 12 (6), 6098–6114. 10.3390/ijerph120606098. 26035660 PMC4483690

[ref2] Freire C.; Vela-Soria F.; Castiello F.; Salamanca-Fernández E.; Quesada-Jiménez R.; López-Alados M. C.; Fernandez M. F.; Olea N. Exposure to Perfluoroalkyl Substances (PFAS) and Association with Thyroid Hormones in Adolescent Males. Int. J. Hyg Environ. Health 2023, 252, 114219 10.1016/j.ijheh.2023.114219. 37451108

[ref3] Rubin I. L.; Nodvin J. T.; Geller R. J.; Teague W. G.; Holtzclaw B. L.; Felner E. I. Environmental Health Disparities: Environmental and Social Impact of Industrial Pollution in a Community—the Model of Anniston, AL. Pediatr. Clin. North Am. 2007, 54 (2), 375–398. 10.1016/j.pcl.2007.01.007. 17448365

[ref4] Naumann A.; Alesio J.; Poonia M.; Bothun G. D. PFAS Fluidize Synthetic and Bacterial Lipid Monolayers Based on Hydrophobicity and Lipid Charge. J. Environ. Chem. Eng. 2022, 10 (2), 107351 10.1016/j.jece.2022.107351. 35463622 PMC9029377

[ref5] Morales-McDevitt M. E.; Becanova J.; Blum A.; Bruton T. A.; Vojta S.; Woodward M.; Lohmann R. The Air That We Breathe: Neutral and Volatile PFAS in Indoor Air. Environ. Sci. Technol. Lett. 2021, 8 (10), 897–902. 10.1021/acs.estlett.1c00481. 35359817 PMC8963212

[ref6] De Silva A. O.; Armitage J. M.; Bruton T. A.; Dassuncao C.; Heiger-Bernays W.; Hu X. C.; Kärrman A.; Kelly B.; Ng C.; Robuck A.; Sun M.; Webster T. F.; Sunderland E. M. PFAS Exposure Pathways for Humans and Wildlife: A Synthesis of Current Knowledge and Key Gaps in Understanding. Environ. Toxicol. Chem. 2021, 40 (3), 631–657. 10.1002/etc.4935. 33201517 PMC7906948

[ref7] Savvaides T.; Koelmel J. P.; Zhou Y.; Lin E. Z.; Stelben P.; Aristizabal-Henao J. J.; Bowden J. A.; Godri Pollitt K. J. Prevalence and Implications of Per- and Polyfluoroalkyl Substances (PFAS) in Settled Dust. Curr. Environ. Health Rep. 2021, 8 (4), 323–335. 10.1007/s40572-021-00326-4. 34985714 PMC11784640

[ref8] Voloshina O. V.; Shirshin E. A.; Lademann J.; Fadeev V. V.; Darvin M. E. Fluorescence Detection of Protein Content in House Dust: The Possible Role of Keratin. Indoor Air 2017, 27 (2), 377–385. 10.1111/ina.12326. 27538819

[ref9] Chen H.; He P.; Rao H.; Wang F.; Liu H.; Yao J. Systematic Investigation of the Toxic Mechanism of PFOA and PFOS on Bovine Serum Albumin by Spectroscopic and Molecular Modeling. Chemosphere 2015, 129, 217–224. 10.1016/j.chemosphere.2014.11.040. 25497588

[ref10] Bischel H. N.; MacManus-Spencer L. A.; Luthy R. G. Noncovalent Interactions of Long-Chain Perfluoroalkyl Acids with Serum Albumin. Environ. Sci. Technol. 2010, 44 (13), 5263–5269. 10.1021/es101334s. 20540534

[ref11] Fedorenko M.; Alesio J.; Fedorenko A.; Slitt A.; Bothun G. D. Dominant Entropic Binding of Perfluoroalkyl Substances (PFASs) to Albumin Protein Revealed by 19F NMR. Chemosphere 2021, 263, 128083 10.1016/j.chemosphere.2020.128083. 33297081 PMC8479757

[ref12] Camdzic D.; Dickman R. A.; Aga D. S. Total and Class-Specific Analysis of per- and Polyfluoroalkyl Substances in Environmental Samples Using Nuclear Magnetic Resonance Spectroscopy. J. Hazard. Mater. Lett. 2021, 2, 100023 10.1016/j.hazl.2021.100023.

[ref13] Foo A. C. Y.; Thompson P. M.; Chen S. H.; Jadi R.; Lupo B.; DeRose E. F.; Arora S.; Placentra V. C.; Premkumar L.; Perera L.; Pedersen L. C.; Martin N.; Mueller G. A. The Mosquito Protein AEG12 Displays Both Cytolytic and Antiviral Properties via a Common Lipid Transfer Mechanism. Proc. Natl. Acad. Sci. U.S.A. 2021, 118 (11), e2019251118 10.1073/pnas.2019251118. 33688047 PMC7980415

[ref14] Dutta S.; Watson B.; Mattoo S.; Rochet J.-C. Calcein Release Assay to Measure Membrane Permeabilization by Recombinant Alpha-Synuclein. Bio-Protoc. 2020, 10 (14), e3690 10.21769/BioProtoc.3690. 32953942 PMC7500579

[ref15] Sørli J. B.; Låg M.; Ekeren L.; Perez-Gil J.; Haug L. S.; Da Silva E.; Matrod M. N.; Gützkow K. B.; Lindeman B. Per- and Polyfluoroalkyl Substances (PFASs) Modify Lung Surfactant Function and pro-Inflammatory Responses in Human Bronchial Epithelial Cells. Toxicol. In Vitro 2020, 62, 104656 10.1016/j.tiv.2019.104656. 31536757

[ref16] Martínez Sarrasague M.; Cimato A.; Rubin De Celis E.; Facorro G. Influence of Serum Protein and Albumin Addition on the Structure and Activity of an Exogenous Pulmonary Surfactant. Respir. Physiol. Neurobiol. 2011, 175 (3), 316–321. 10.1016/j.resp.2010.12.009. 21185407

[ref17] Zuo Y. Y.; Tadayyon S. M.; Keating E.; Zhao L.; Veldhuizen R. A. W.; Petersen N. O.; Amrein M. W.; Possmayer F. Atomic Force Microscopy Studies of Functional and Dysfunctional Pulmonary Surfactant Films, II: Albumin-Inhibited Pulmonary Surfactant Films and the Effect of SP-A. Biophys. J. 2008, 95 (6), 2779–2791. 10.1529/biophysj.108.130732. 18539636 PMC2527281

[ref18] Toimil P.; Prieto G.; Jr J. M.; Trillo J. M.; Sarmiento F. Interaction of Human Serum Albumin with Monofluorinated Phospholipid Monolayers. J. Colloid Interface Sci. 2012, 388 (1), 162–169. 10.1016/j.jcis.2012.08.035. 23010317

[ref19] Blume A.; Kerth A. Peptide and Protein Binding to Lipid Monolayers Studied by FT-IRRA Spectroscopy. Biochim. Biophys. Acta, Biomembr. 2013, 1828 (10), 2294–2305. 10.1016/j.bbamem.2013.04.014. 23816442

[ref20] Oeo-Santos C.; Lopez-Rodriguez J. C.; Garcia-Mouton C.; San Segundo-Acosta P.; Jurado A.; Moreno-Aguilar C.; Garcia-Alvarez B.; Perez-Gil J.; Villalba M.; Barderas R.; Cruz A. Biophysical and Biological Impact on the Structure and IgE- Binding of the Interaction of the Olive Pollen Allergen Ole e 7 with Lipids. Biochim. Biophys. Acta, Biomembr. 2020, 1862 (6), 183258 10.1016/j.bbamem.2020.183258. 32142819

[ref21] López-Rodríguez J. C.; Barderas R.; Echaide M.; Pérez-Gil J.; Villalba M.; Batanero E.; Cruz A. Surface Activity as a Crucial Factor of the Biological Actions of Ole e 1, the Main Aeroallergen of Olive Tree (Olea Europaea) Pollen. Langmuir 2016, 32 (42), 11055–11062. 10.1021/acs.langmuir.6b02831. 27723354

[ref22] Schmidt R.; Meier U.; Markart P.; Grimminger F.; Velcovsky H. G.; Morr H.; Seeger W.; Günther A. Altered Fatty Acid Composition of Lung Surfactant Phospholipids in Interstitial Lung Disease. Am. J. Physiol.: Lung Cell. Mol. Physiol. 2002, 283 (5), 1079–1085. 10.1152/ajplung.00484.2001. 12376361

[ref23] Meyer K. C.; Sharma A.; Brown R.; Weatherly M.; Moya F. R.; Lewandoski J.; Zimmerman J. J. Function and Composition of Pulmonary Surfactant and Surfactant-Derived Fatty Acid Profiles Are Altered in Young Adults With Cystic Fibrosis. Chest 2000, 118 (1), 164–174. 10.1378/chest.118.1.164. 10893374

[ref24] Hite R. D.; Seeds M. C.; Jacinto R. B.; Balasubramanian R.; Waite M.; Bass D. Hydrolysis of Surfactant-Associated Phosphatidylcholine by Mammalian Secretory Phospholipases A2. Am. J. Physiol.: Lung Cell. Mol. Physiol. 1998, 275 (4), L740–L747. 10.1152/ajplung.1998.275.4.l740. 9755106

[ref25] Hite R. D.; Seeds M. C.; Jacinto R. B.; Grier B. L.; Waite B. M.; Bass D. A. Lysophospholipid and Fatty Acid Inhibition of Pulmonary Surfactant: Non-Enzymatic Models of Phospholipase A2 Surfactant Hydrolysis. Biochim. Biophys. Acta, Biomembr. 2005, 1720 (1–2), 14–21. 10.1016/j.bbamem.2005.10.014. 16376294

[ref26] Moses D.; Holm B. A.; Spitale P.; Liu M.; Enhorning G. Inhibition of Pulmonary Surfactant Function by Meconium. Am. J. Obstet. Gynecol. 1991, 164 (2), 477–481. 10.1016/S0002-9378(11)80003-7. 1992687

[ref27] Kinkaid A.; Wilton D. C. Comparison of the Catalytic Properties of Phospholipase A2 from Pancreas and Venom Using a Continuous Fluorescence Displacement Assay. Biochem. J. 1991, 278 (3), 843–848. 10.1042/bj2780843. 1898370 PMC1151423

[ref28] Duchez A. C.; Boudreau L. H.; Naika G. S.; Rousseau M.; Cloutier N.; Levesque T.; Gelb M. H.; Boilard E. Respective contribution of cytosolic phospholipase A2α and secreted phospholipase A2 IIA to inflammation and eicosanoid production in arthritis. Prostaglandins Other Lipid Mediators 2019, 143 (May), 106340 10.1016/j.prostaglandins.2019.106340. 31129176

[ref29] Uozumi N.; Kume K.; Nagase T.; Nakatani N.; Ishii S.; Tashiro F.; Komagata Y.; Maki K.; Ikuta K.; Ouchi Y.; Miyazaki J. I.; Shimizu T. Role of Cytosolic Phospholipase A2 in Allergic Response and Parturition. Nature 1997, 390 (6660), 618–622. 10.1038/37622. 9403692

[ref30] Xie W.; Kania-Korwel I.; Bummer P. M.; Lehmler H.-J. Effect of Potassium Perfluorooctanesulfonate, Perfluorooctanoate and Octanesulfonate on the Phase Transition of Dipalmitoylphosphatidylcholine (DPPC) Bilayers. Biochim. Biophys. Acta, Biomembr. 2007, 1768 (5), 1299–1308. 10.1016/j.bbamem.2007.02.003. PMC1993895 17349969

[ref31] Wang H.; Zhang X.; Liu Y.; Liu J. Stabilization of Liposomes by Perfluorinated Compounds. ACS Omega 2018, 3 (11), 15353–15360. 10.1021/acsomega.8b02448. 30556004 PMC6288781

[ref32] Qin W.; Henneberger L.; Huchthausen J.; König M.; Escher B. I. Role of Bioavailability and Protein Binding of Four Anionic Perfluoroalkyl Substances in Cell-Based Bioassays for Quantitative in Vitro to in Vivo Extrapolations. Environ. Int. 2023, 173, 107857 10.1016/j.envint.2023.107857. 36881956

[ref33] Wang M.; Gauthier A.; Daley L. A.; Dial K.; Wu J.; Woo J.; Lin M.; Ashby C.; Mantell L. L. The Role of HMGB1, a Nuclear Damage-Associated Molecular Pattern Molecule, in the Pathogenesis of Lung Diseases. Antioxid. Redox Signaling 2019, 31 (13), 954–993. 10.1089/ars.2019.7818. PMC6765066 31184204

[ref34] Suwara M. I.; Green N. J.; Borthwick L. A.; Mann J.; Mayer-Barber K. D.; Barron L.; Corris P. A.; Farrow S. N.; Wynn T. A.; Fisher A. J.; Mann D. A. IL-1α released from damaged epithelial cells is sufficient and essential to trigger inflammatory responses in human lung fibroblasts. Mucosal Immunol. 2014, 7 (3), 684–693. 10.1038/mi.2013.87. 24172847 PMC3931585

[ref35] Fagotti A.; Lucentini L.; Simoncelli F.; La Porta G.; Brustenga L.; Bizzarri I.; Trio S.; Isidori C.; Di Rosa I.; Di Cara G. HSP70 Upregulation in Nasal Mucosa of Symptomatic Children with Allergic Rhinitis and Potential Risk of Asthma Development. Sci. Rep. 2022, 12 (1), 14104 10.1038/s41598-022-18443-x. 35982171 PMC9388484

[ref36] Syrbu S.; Thrall R. S.; Smilowitz H. M. Sequential Appearance of Inflammatory Mediators in Rat Bronchoalveolar Lavage Fluid after Oleic Acid-Induced Lung Injury. Exp. Lung Res. 1996, 22 (1), 33–49. 10.3109/01902149609074016. 8838134

[ref37] Toborek M.; Lee Y. W.; Garrido R.; Kaiser S.; Hennig B. Unsaturated fatty acids selectively induce an inflammatory environment in human endothelial cells. Am. J. Clin. Nutr. 2002, 75 (1), 119–125. 10.1093/ajcn/75.1.119. 11756069

[ref38] Foo A. C. Y.; Lafont A. P.; Mueller G. A. Expanding the Antiviral Potential of the Mosquito Lipid-Transfer Protein AEG12 Against SARS-CoV-2 Using Hydrophobic Antiviral Ligands. FEBS Lett. 2022, 596 (19), 2555–2565. 10.1002/1873-3468.14456. 35891619 PMC9353291

[ref39] Larsen M. T.; Kuhlmann M.; Hvam M. L.; Howard K. A. Albumin-Based Drug Delivery: Harnessing Nature to Cure Disease. Mol. Cell. Ther. 2016, 4 (1), 3 10.1186/s40591-016-0048-8. 26925240 PMC4769556

[ref40] Hufnagl K.; Afify S. M.; Braun N.; Wagner S.; Wallner M.; Hauser M.; Wiederstein M.; Gadermaier G.; Wildner S.; Redegeld F. A.; Blokhuis B. R.; Hofstetter G.; Pali-Schöll I.; Roth-Walter F.; Pacios L. F.; Jensen-Jarolim E. Retinoic Acid-Loading of the Major Birch Pollen Allergen Bet v 1 May Improve Specific Allergen Immunotherapy: In Silico, in Vitro and in Vivo Data in BALB/c Mice. Allergy Eur. J. Allergy Clin. Immunol. 2020, 75 (8), 2073–2077. 10.1111/all.14259. PMC7522679 32141090

[ref41] Hernandez E. T.; Koo B.; Sofen L. E.; Amin R.; Togashi R. K.; Lall A. I.; Gisch D. J.; Kern B. J.; Rickard M. A.; Francis M. B. Proteins as Adsorbents for PFAS Removal from Water. Environ. Sci.: Water Res. Technol. 2022, 8 (6), 1188–1194. 10.1039/D1EW00501D.

[ref42] Voltolini S.; Spigno F.; Cioè A.; Cagnati P.; Bignardi D.; Minale P. Bovine Serum Albumin: A Double Allergy Risk. Eur. Ann. Allergy Clin. Immunol. 2013, 45 (4), 144–147. 24067340

[ref43] Choi G.-S.; Kim J.-H.; Lee H.-N.; Sung J.-M.; Lee J.-W.; Park H.-S. Occupational Asthma Caused by Inhalation of Bovine Serum Albumin Powder. Allergy, Asthma Immunol. Res. 2009, 1 (1), 45 10.4168/aair.2009.1.1.45. 20224670 PMC2831573

[ref44] Chen M.-C.; Tai J. W.; Wu C.-J. Induction of Airway Hypersensitivity to Ovalbumin and Dust Mite Allergens as Mouse Models of Allergic Asthma. In Animal Models of Allergic Disease: Methods and Protocols; Nagamoto-Combs K., Ed.; Springer US: New York, NY, 2021; pp 101–114. 10.1007/978-1-0716-1001-5_8 33226590

[ref45] Foo A. C. Y.; Mueller G. A. Abundance and Stability as Common Properties of Allergens. Front. Allergy 2021, 2, 769728 10.3389/falgy.2021.769728. 35386965 PMC8974735

