# Supplementary material for: Albumin Proteins as Delivery Vehicles for PFAS Contaminants into Respiratory Membranes
Source: ACS Omega. 2023 Nov 9;8(46):44036–43. doi: 10.1021/acsomega.3c06239 (PMC10666230; doi:10.1021/acsomega.3c06239)
Supplement: Supplementary file 1 — ao3c06239_si_001.pdf [file ao3c06239_si_001.pdf]

Supporting Information

Albumin proteins as delivery vehicles for PFAS contaminants into respiratory membranes

Evan S. Pye, Shannon E. Wallace, D. Gerrard Marangoni, and Alexander C.Y. Foo*

St. Francis Xavier University, Dept. of Chemistry. 2321 Notre Dame Ave. Antigonish, NS, Canada. B2G 2W5. E-mail: afoo@stfx.ca

S1

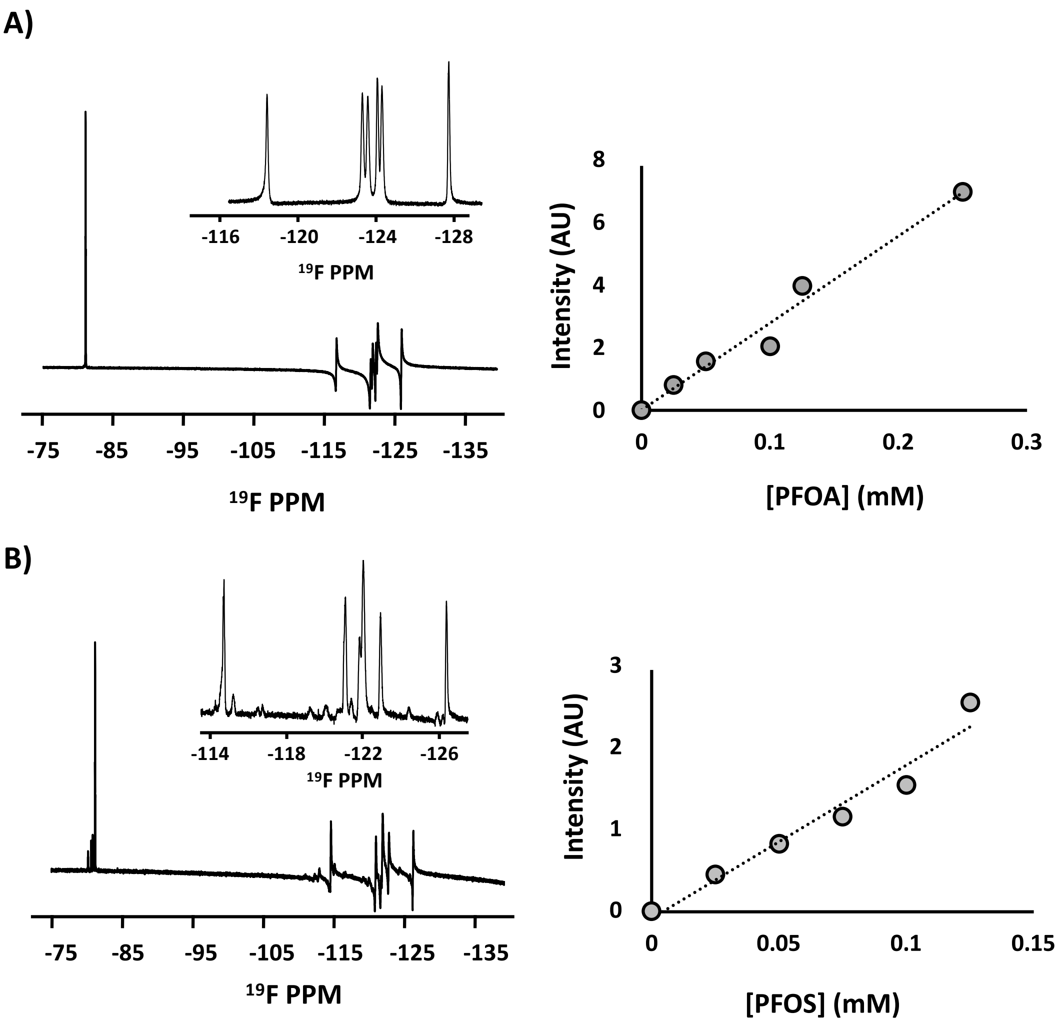

S1: ^{19}F-NMR Spectra of PFOA and PFOS. Representative ^{19}F-NMR spectra for PFOA (A) and PFOS (B). Based on previous studies, we assigned the peaks on the far right (~-80 ppm) to the terminal CF_3 fluorine nuclei due to their separation from the other CF_2 peaks, which are clustered around –114 to -126 ppm.[1] A detailed view of the CF_2 peaks is shown in the inset. The standard curves (in AU, or arbitrary units) obtained for both PFOA and PFOS is shown on the right.

The NMR spectra for PFOS show some minor species, perhaps reflecting the reduced solubility of PFOS compared to PFOA, resulting in the formation of larger aggregates. However, the linear nature of the standard curve suggests that this did not notably impact quantitation.

S2

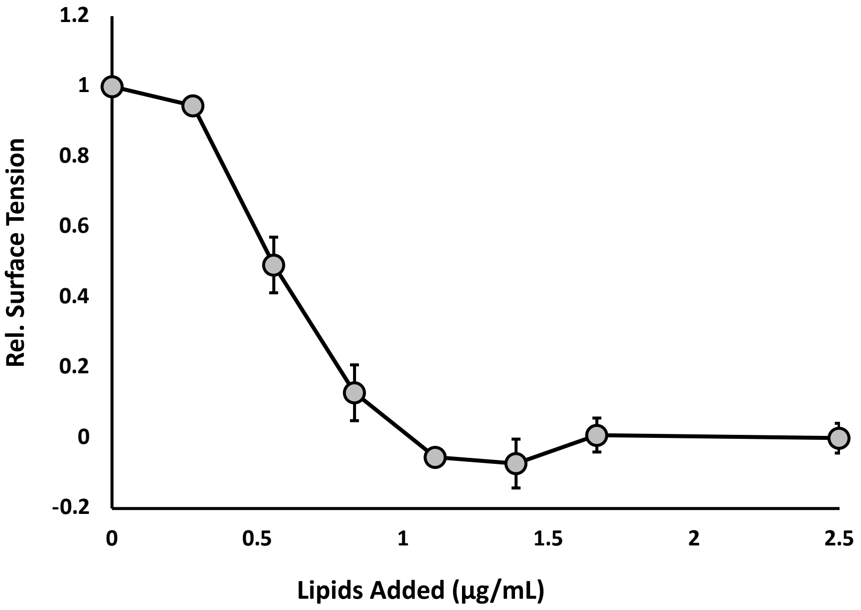

S2: Confirming the presence of a complete lipid film. Varying amounts of DPPC lipids were deposited onto the surface of an aqueous sub-phase in order to generate a lipid film as discussed in the Materials and Methods section of this work. The relative change in surface tension was plotted against the amount of lipids added (µg lipid per mL of aqueous phase) to generate a sigmoidal curve. Minimum surface tension occurs upon formation of a complete monolayer, which corresponds to a lipid loading of ~1.5 µg/mL under our experimental conditions.

References:

(1) Camdzic, D.; Dickman, R. A.; Aga, D. S. Total and Class-Specific Analysis of per- and Polyfluoroalkyl Substances in Environmental Samples Using Nuclear Magnetic Resonance Spectroscopy. *J. Hazard. Mater. Lett.* **2021**, *2*, 100023. https://doi.org/10.1016/j.hazl.2021.100023.
